# Vaccinomics to Design a Multiepitope Vaccine against *Legionella pneumophila*

**DOI:** 10.1155/2022/4975721

**Published:** 2022-09-17

**Authors:** Ahitsham Umar, Sadia Liaquat, Israr Fatima, Abdur Rehman, Danish Rasool, Abdulrahman Alshammari, Metab Alharbi, Muhammad Shahid Riaz Rajoka, Mohsin Khurshid, Usman Ali Ashfaq, Asma Haque

**Affiliations:** ^1^Department of Bioinformatics and Biotechnology, Government College University Faisalabad, Faisalabad 38000, Pakistan; ^2^Department of Pharmacology and Toxicology, College of Pharmacy, King Saud University, P.O. Box 2455, Riyadh 11451, Saudi Arabia; ^3^Laboratory of Animal Food Function, Graduate School of Agricultural Science, Tohoku University, Sendai 980-8572, Japan; ^4^Department of Microbiology, Government College University Faisalabad, Faisalabad 38000, Pakistan

## Abstract

*Legionella pneumophila* is found in the natural aquatic environment and can resist a wide range of environmental conditions. There are around fifty species of Legionella, at least twenty-four of which are directly linked to infections in humans. *L. pneumophila* is the cause of Legionnaires' disease, a potentially lethal form of pneumonia. By blocking phagosome-lysosome fusion, *L. pneumophila* lives and proliferates inside macrophages. For this disease, there is presently no authorized multiepitope vaccine available. For the multi-epitope-based vaccine (MEBV), the best antigenic candidates were identified using immunoinformatics and subtractive proteomic techniques. Several immunoinformatics methods were utilized to predict B and T cell epitopes from vaccine candidate proteins. To construct an *in silico* vaccine, epitopes (07 CTL, 03 HTL, and 07 LBL) were carefully selected and docked with MHC molecules (MHC-I and MHC-II) and human TLR4 molecules. To increase the immunological response, the vaccine was combined with a 50S ribosomal adjuvant. To maximize vaccine protein expression, MEBV was cloned and reverse-translated in *Escherichia coli*. To prove the MEBV's efficacy, more experimental validation is required. After its development, the resulting vaccine is greatly hoped to aid in the prevention of *L. pneumophila* infections.

## 1. Introduction

Legionnaires' disease is a severe form of pneumonia caused by *L. pneumophila*, aerobic Gram-negative, motile, and rod-shaped bacteria of the proteobacterial lineage. These bacteria are primarily found in artificial aquatic environments [1]. An episomal and chromosomal 45 kb pair region, selective expansions of key gene families, genes for unexpected metabolic pathways, and previously recognized putative virulence factors make up its genome. Intracellular pathogens exploit the iCM/dot type secretion system to deliver effector proteins to host cells, altering phagocytic vacuole destiny by inhibiting phagosome-lysosome fusion, vacuole acidification, and recruitment of vesicles with endoplasmic reticulum-like characteristics [2–4]. Virulence levels of Legionella species have different ranges in nonpathogenic *L. pneumophila* [5, 6].

Lung infection is caused by inhaling microdroplets as well as aerosols from water infected through Legionella. Though these bacteria are common in natural water systems, the sickness is caused by higher populations in artificial water systems, which are more likely to produce aerosols that host the bacteria. Premise plumbing, misters, showers, and cooling towers are examples of aerosol-producing designed water systems [7]. Throughout the SARS-CoV2 pandemic, the situation has become even more severe, as massive worldwide “stay-at-home” orders have made a conducive atmosphere for the growth of Legionella and propagation [8]. In reaction to the new coronavirus epidemic, the directives have resulted in the closure of buildings, institutions, and offices. Water usage and flow have been curtailed due to the widespread closures and “shut-downs,” resulting in water stagnation [9]. In 1976, Philadelphia was the site of the first big outbreak of Legionnaires' disease (LD). Since then, many attacks and occasional cases have been documented, most linked to *Legionella pneumophila* bacteria [10]. According to the US National Academies of Sciences, Engineering, and Medicine (NASEM), Legionella is five times higher than its 20-year previous incidence [11]. The total number of cases in both the United States and Canada is thought to be significantly increased [1, 12].

For Legionella detection, two methods are utilized: ISO 2017 culture (AFNOR NF T90-431, ISO 11731) and qPCR (AFNOR NF T90-471, ISO/TS 12869) [13, 14]. The culture method is time-demanding, sensitive to sample selection, and prone to false-negative results because of interfering flora and viable but nonculturable (VBNC) cells [15]. The qPCR approach is more sensitive and rapid, but it risks overestimating Lp levels because it cannot differentiate live from nonviable bacteria [16]. It also necessitates additional processing and elimination processes to remove chemicals from complex matrices that could interfere with qPCR responses [16, 17]. The use of surface plasmon resonance imaging (SPRi) biosensors can solve several issues that contemporary technologies pose. Unlike present approaches, real-time detection could be carried out by using SPRI technology, allowing complex samples, and detecting surface regeneration, all of which would avoid several processing stages and ensure the system's convenient operation at a cheaper cost [18, 19].

There is presently no approved *L. pneumophila* medication vaccination available, while a few investigational medications are undergoing clinical studies. Vaccination is the most efficient way to avoid viral infections. The availability of genetic data, advanced software, and immunological data sets now make it much easier for researchers to find efficient active subunit vaccines that could be manufactured using epitopes of infection proteins [20–22].

Antigenic proteins that may be anticipated for T cells and B cells using their major histocompatibility complex (MHC) alleles were found in the proteome of *L. pneumophila*. Antigenicity, conservation, and worldwide coverage of expected epitopes have all been explored by linking the most promising and interacting epitopes, and by adding an adjuvant, a multi-epitope-based vaccine (MEBV) was constructed. Since MEBVs are more cost-effective than conventional single-epitope vaccines, they are also more stable specific and time-efficient. Due to the inclusion of both T cell and B cell epitopes, they are supposed to elicit large humoral and cellular immunological responses simultaneously [23]. Several *in silico* techniques were used to validate the antigenicity, allergenicity, immunogenicity, toxicity, structural stability/flexibility, and physicochemical properties of developed MEBVs. The usage of computational methods and molecular dynamic simulation was employed to evaluate the stability and interaction of MEBV with human receptors. Moreover, *in silico* cloning was performed to demonstrate expression profiling using MEBV codons optimized for *E. coli*.

There is currently no way to either cure the condition or prevent it via drugs or immunizations. This study used subtractive proteomics, reverse vaccinology, and immunoinformatics as the research tools to identify key targets of *L. pneumophila* that may be exploited in the development of vaccines to regulate humoral and cellular immune responses. These targets may be exploited in the development of vaccines. Because of this discovery, scientists now could perform research experiments toward the development of a vaccine that can protect against *L. pneumophila* as well as the many other viruses that are resistant to antibiotics. This approach has been used to predict various MEBVs for different viral infections.

## 2. Methodology

### 2.1. Retrieval of Proteome Analysis

The whole proteome of the *L. pneumophila* strain was extracted in FASTA format using UniProt [24]. The Geptop 2.0 server was used to find essential proteins [25]. Human proteins should not be used as vaccine candidates to prevent an autoimmune response. Nonhomologous proteins were predicted using BLASTp, and in this general sequence, identification and similarity search with different proteins was done [26]. The outer membrane proteins were chosen by cellular localization using PSORTb 3.0.3 [27]. Since immunogenicity is a measure of someone's capacity for quick response and generating an effective immune response to an antigen, peptide-based vaccines were created using more antigenic proteins. The Vaxijen 2.0 server was used to evaluate the antigenicity of all *L. pneumophila* proteins with a 0.5 threshold [28]. This server has a 70-89 percent accuracy when employing the autocross covariance transformation algorithm [28]. To predict the transmembrane helix, the TMHMM v-2.0 server was employed [29].

### 2.2. CTL Epitope Selection and Evaluation

Cytotoxic T cells are crucial in identifying specific antigens and the correct design of CTL epitopes, which are vital for vaccine development. Most importantly, compared to wet laboratory tests, it reduces the time and cost of predicting epitopes [30].CTL epitopes were determined using Immune Epitope Database's MHC-I binding tool (IEDB) [31]. Multiple options used to run the queries that have multiple tools that are linked within its resource options like MHC-I and MHC-II could be predicted by using the links given in the epitope analysis resource option box; B cell prediction tool links are given in the B cell prediction toolbox, same as population coverage could be predicted by epitope analysis tool options.

Because a lower percentile rank suggests better affinity, the percentile rank was set to 2. Highly antigenic, immunogenic, and nontoxic CTL epitopes were selected to develop vaccines. The IEDB database was used to calculate the immunogenicity of the epitopes [31]. Toxicity and antigenicity were determined using the Toxin Pred and Vaxijen servers [32, 33]. The Toxin Pred server uses a quantitative matrix and machine learning technique to analyze peptide characteristics. In this tool, the SVM method by default *E* value cutoff value was used that was 10 to predict if the epitope is toxic or nontoxic. Allergenicity was determined with the help of AllergenFP, which has an accuracy of 88.9% [34].

### 2.3. Selection and Analysis of Helper T Lymphocyte (HTL) Epitopes

HTL cells are the most important cells in the adaptive immune response because they turn on CTL cells, which kill pathogens, macrophages, which eat pathogens, and B cells, which make antibodies [35]. It is, therefore, crucial to involve helper T cell epitopes for a healthy immune response. HTL can produce interleukin-4 (IL-4) and IL-10 as well as interferon-gamma (IFN-*γ*), which causes immune cells including macrophages and cytotoxic T cells to become activated [36]. As a result, HTL epitopes that produce cytokines are crucial for vaccine development. The IEDB employed its MHC-II binding tool to predict HTL epitopes (15-mer) of target proteins [31]. This tool was used to predict IFN-gamma-inducing regions in the protein of interest. The SVM method is used, and positively scored epitopes were selected for further process.

In SOPMA, by applying motif scan and SVM approach, overlapping HTL sequence peptides were found against the query protein using the IFN-*γ* epitope server [37]. The interleukin-4 (IL-4) web server allows design, discovery, and peptide map to genuinely produce IL-4 with the threshold of 0.2, which is significant for subunit vaccines [38]. At a threshold value of 0.3, IL-10 Pred was also employed to predict the inducing qualities [39].

### 2.4. LBL Epitope Identification and Evaluation

The surface receptor identified B cell epitopes, which create antigen-related immunoglobulins. As a result, developing a B cell epitope vaccination would be critical for adaptive immunity [40]. ABCPred, an internet-based server based on neural networks, identified LEPS B cells with the highest accuracy of 75 percent [41]. AllergenFP v1.0 was utilized to test the allergenicity of the query sequence. ToxinPred retrieved toxicity, and VaxiJen v2.0 servers retrieved antigenicity of anticipated B cell epitopes [28, 32, 34].

### 2.5. Population Coverage Analysis

In different ethnic groups, distinct HLA alleles and their expression are distributed at varied frequencies. The distribution of HLA alleles around the globe is thus critical for the creation of multiepitope vaccines [42]. The primary objective of these population coverage studies determines if the candidates selected were suitable for a broad group of individuals [43]. Population coverage of the specified epitopes and their HLA alleles was determined using the IEDB's population coverage server [44].

### 2.6. Vaccine's Mapping

The selected CTL, HTL, and LBL epitopes were linked and formed a fusion peptide using different linkers like AAY, KK, and GPGPG and 50S ribosomal protein L7/L12 as adjuvant. By connecting, coupling adjuvants to the sequence may enhance immunogenicity and long-lasting immune response. As a result, the EAAAK linker was used to attach the TLR4 against (RS-09; Sequence: APPHALS) adjuvant to the CTL epitope. For each epitope to function properly, a linker must be employed to connect two epitopes.

### 2.7. Structural Analysis

The vaccine was first examined using BLASTp to see if it was identical to the human proteome [45]. The vaccine construct's physiochemical features were assessed using the ProtParam web server [46]. The molecular weight, extension coefficient, isoelectric point, and half-life were all computed by ProtParam. The Vaxijen server was utilized to assess v antigenicity [31, 33]. Another crucial step was to use the AllerTOP service to calculate the vaccine design's allergenicity (allergen or nonallergen) [47]. The SOPMA tool, which is critical for predicting protein folding, predicts the vaccine's 2D structure. In this tool, the number of confirmational states was 4 (helix, sheet, turn, and coils); the similarity threshold was set to be default as 8. SOPMA's secondary structure prediction is 69.5 percent accurate, with a three-state structure (*α*-helix, *β*-sheet, and coil) description [48]. The ProtParam tool was used to evaluate the physiochemical properties of our vaccine. The SOLpro is a useful tool used for predicting vaccine solubility in the final stages of vaccine development, with a prediction accuracy of 74% and several runs of 10-fold cross-validation [49].

### 2.8. Prediction of Tertiary Structure, Confirmation, and Refinement

The amino acid sequence can be used to construct a 3D protein model using computer techniques. The I-TASSER suit was used to model the MEBV in three dimensions (3D) [50]. It takes structural templates from the protein database through multiple threading approaches and then predicts the structure through iterative template-based fragment assembly simulation.

The vaccine model was refined using the GalaxyRefine web server after it was predicted [51]. The GalaxyRefine server reshapes the side chain before performing structural reassembling and overall structural assessment using molecular dynamics. As the CASP refinement category demonstrates, it is extremely helpful in increasing the quality of local structures. The Ramachandran plot was used to determine which phi and psi dihedral angle preferences of amino acid residues were energetically allowed or banned [52]. Furthermore, ProSA-web calculates special requirements to verify the 3D structures obtained from NMR spectroscopy, X-ray, and theoretical calculations [53]. The ERRAT program predicted the tertiary structure of the protein and assigned a quality factor based on nonbonded atomic interactions within the protein [54].

### 2.9. B Cell Epitope Screening

The ABCpred server was used to identify conformational and linear B cell epitopes [41, 55]. The ABCpred server set the length and threshold of the MEBV amino acid sequence to 14 and 0.5, respectively. The MEBV's tertiary structure was also entered into the ElliPro program using the default parameters.

### 2.10. Docking of TLR4 Receptor with Constructed Vaccine Disulphide

The constructed vaccine candidate ultimately interacts with host immune cells to elicit an efficient immune response. HADDOCK 2.2 is utilized to dock MEBV with human Toll-like receptors (TLR4) and MHC molecules (MHC-I and MHC-II) [56]. HADDOCK is a high ambiguity docking program that may use data from the interface zone between molecular components and their orientations. *C* port values of both protein and residue have to paste in given boxes as well as structures of both have to attach there. In output, we received the docking score, cluster size, RMSD values, and Van-der-Waals energy values.

In contrast to other docking tools, the HADDOCK V2.2 step allows overall conformational changes involving the modifications in the backbone. Docking multimodel NMR structures and other Protein Data Bank (PDB) structures are also possible with HADDOCK [57]. Crystal structures of TLR4 (ID: 4G8A), TLR2 (ID: 2Z7X), MHC-I (ID: 1I1Y), and MHC-II (ID: 1KG0) were retrieved from the Protein Data Bank (PDB) (ID: 1KG0) [58–61]. Analysis of docked complexes in the PDBsum database and imaging of the docked complexes with the PyMOL molecular visualization system leads to the discovery of interactions [62, 63]. PDBsum is an online web server that depicts a wide variety of information about a macromolecular structure in a PDB. This includes PROMOTIF structural analysis, images of the structure, PROCHECK results, and schematic illustrations of protein-DNA and protein-ligand interactions.

### 2.11. Molecular Dynamic Simulation

Molecular dynamics plays an important role in confirming and determining the stability of proteins and protein complexes in all *in silico* studies. Analyzing the modes of essential proteins can measure protein stability [64, 65]. The iMODs (internal coordinate normal mode analysis) server was utilized to describe joint-protein mobility in inner coordinates [66]. Internal complex movements were measured using this site. The value of the standard mode was determined by the stiffness of motion. When the eigenvalue is low, it aids in the deformation of structures that have a certain energy.

### 2.12. Immune Stimulation

The C-IMMSIM web server evaluated the immunological profile of the MEBV finalized candidate. The C-IMMSIM is a flexible tool that uses a position-specific scoring matrix (PSSM) to forecast the peptide interactions for immune response. The C-IMMSIM web server is commonly used in immune informatics investigations and provides accurate findings in terms of vaccination strategy [67–69]. The simulation was carried out in 1000 steps over four weeks, with two dosages administered.

### 2.13. In Silico Cloning and Optimization of Codons

A codon optimization technique can help the host increase the expression of foreign genes. Codon usage fluctuates from organism to organism, and this variation in codon usage may lead to a low foreign gene expression rate. The vaccine sequence was codon adapted using the JCat tool, ensuring the codon usage per *E. coli* K12 strain [70]. Finally, an *E. coli* vector was used to clone the modified nucleotide sequence of pET30a (+) by employing Snap Gene v5.0.8 software [71]. The pET30a cloning vector was used in this project because it has similar restriction sites regarding MEBV, and it has a high expression level than pET28a. pET28a had no similar restriction sites according to the vaccine construct [72–74].

## 3. Results

### 3.1. Protein Selection

To identify the most promising candidates for *Legionella pneumophila* MEBV design, this study used a subtractive proteomic technique. It slowly gets rid of less desirable proteins from the complete proteome of *L. pneumophila*. “ATCC 33152/DSM 7513” is the name of the *L. pneumophila* strain that was downloaded from UniProt. It has 2889 proteins (Accession no.: UP000000609). The proteome was filtered using CD-HIT with a threshold of 80% (0.8). Nonparalogous proteins were removed from further analysis using the CD-HIT suite. The Geptop server was used to identify essential proteins. The essentiality score cutoff was set to be 0.24. Only 398 essential proteins were found, and their similarity with the human proteome was confirmed (Taxonomic id: 9606). To avoid an autoimmune reaction that attacks and destroys its cells after identifying them as alien particles, it is critical to get rid of human homologs. To avoid a situation like this, homologous proteins should be avoided. The CELLO server projected that those 379 essential proteins would be found in the cytoplasm, 11 in the outer membrane region and 8 in extracellular locations. For further study, the cytoplasmic proteins were eliminated, while the remaining proteins were selected. Six of the proteins were chosen for the epitope's prediction and further procedure (Table 1).

### 3.2. CTL Epitope Selection and Evaluation

From the L. pneumophila target protein, thirty-one CTL epitopes (12-mer) were identified (Table S1). With the help of this method, the top seven epitopes with the highest immunogenicity, antigenicity, and nonallergenicity as well as nontoxicity were discovered for vaccine development (Table 2). There was a total of 6 unique HTL epitopes chosen for vaccine construction (Table S2). The top 3 epitopes were finalized for vaccine construction based on their cytokine capacity, and only these 3 fulfill the following properties of antigenicity, allergenicity, and toxicity (Table 3). Similarly, after analyzing the allergenicity, toxicity, and immunogenicity of 35 LBL epitopes (Table S3), a total of 7 epitopes were selected for vaccine manufacture (Table 4).

### 3.3. Population Coverage

**Figure 1:**
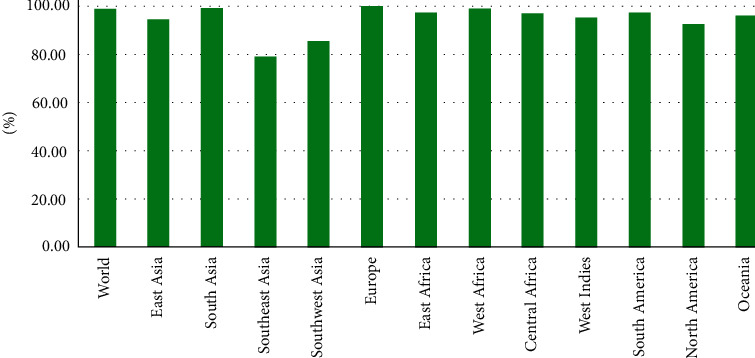
Worldwide population coverage analysis of selected epitopes.

In the development of vaccines, population coverage is a critical aspect. The whole population coverage of selected MHC-I and MHC-II epitopes with corresponding HLA alleles was assessed in this study. The overall coverage of the selected epitopes was calculated to be 87% of the world's population. North America was discovered to have the highest population coverage, at 99.99 percent. According to our research, the chosen epitopes are expected to be great vaccine candidates (Figure 1).

### 3.4. Construction of Vaccine

**Figure 2:**
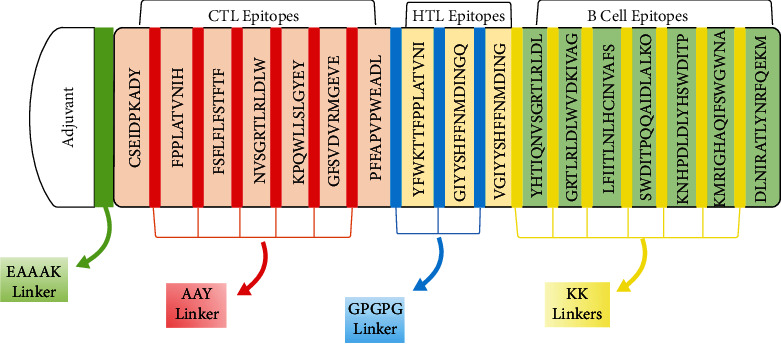
Constructed vaccine figure with adjuvants and linkers. Each color represents the specific linker.

For vaccine construction, all the selected epitopes were utilized. All CTL, HTL, and LBL epitopes were attached using AAY, KK, and GPGPG linkers. These linkers were chosen because they aid in vaccination and epitope presentation while preventing junctional epitope formation [75, 76]. With the first CTL epitopes, the 50S ribosomal protein L7/L12 (124 residues) was employed as an adjuvant, along with the EAAAK linker. The EAAAK linker improves structural stability with effective division and reduces interaction with neighboring protein regions [77]. The final 419 amino acid vaccine design demonstrates how different epitopes and linkers were used to develop a successful vaccination (Figure 2).

### 3.5. Physiochemical and Immunogenic Profiling

The manufactured vaccine's immunogenicity and physicochemical properties are investigated further. When the similarity of the made vaccine to the human proteome was compared, it was found that no two human proteomes are the same. As a result of these analyses, our vaccine proved to be highly antigenic, nonallergenic, and nontoxic. ProtParam was used to evaluate the physicochemical properties of the compounds. The final construct had 7.61pI 46284.37 kDa MW, respectively. The GRAVY was calculated to be -0.065 and has a half-life *in vitro* of thirty hours, in vivo of more than twenty hours (yeast), and in vivo of more than ten hours (*E. coli*). SOLpro results indicated soluble with probability of 0.937616. All these characteristics suggest that *L. pneumophila is a suitable vaccine candidate.*

### 3.6. Structural Evaluation

The following characteristics also support *L. pneumophila* as a promising vaccine candidate. The alpha-helix contains 155 amino acids, which account for 36.99 percent of the sequence, 110 amino acids in extended chains, which account for 26.25 percent, and 108 amino acids in coils, which account for 25.78 percent of the vaccine's construct.

### 3.7. Prediction of Tertiary Structure, Refinement, and Validation

**Figure 3:**
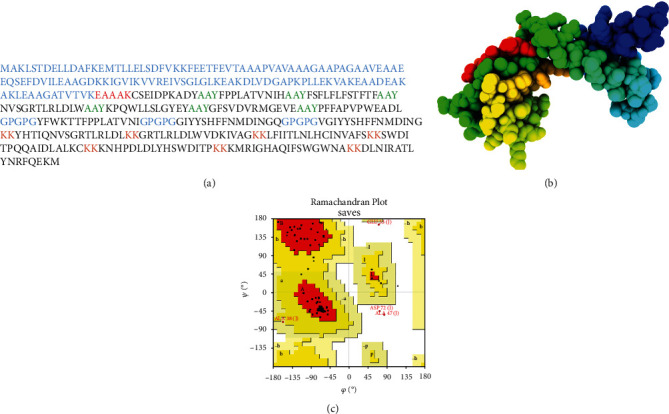
(a–c) Vaccine construct sequence along with predicted structure and structure evaluation analysis: (a) depicts the sequence of the vaccine after addition of different linkers and an adjuvant; (b) is the predicted structure of the vaccine using I-TASSER server; (c) describes the conformational analysis of the structure, and the red zones in that plot indicated the favored regions and the number of residues in it.

3D structure prediction of our vaccine was done through an online server I-TASSER. *C*-score was measured as -1.66 in the results. The structure was modified and refined by utilizing the GalaxyRefine web server. After that, analysis of the modeled structure was done through the Ramachandran plot, which indicated the best values of the favored region about 92.7 percent, 3.5 percent in the favored region, and 2.7 percent in the outer region. *Z*-score was calculated as 0.395, and ERRAT analysis exhibited a score of 95. These results proved our refined model to be excellent (Figures 3(a)–3(c).

### 3.8. Selection of B Cell Epitopes

B lymphocytes generate antibodies, which result in humoral immunity [78]. Therefore, the vaccination must have excellent B cell epitope domains. Using default settings, it was utilized to predict 24 linear-continuous and 3 conformational-discontinuous vaccine constructs using ABCPred 2.0. The conformational B cell epitopes in the vaccine design were visualized using PyMOL v.1.3, a molecular graphic system.

### 3.9. Molecular Docking

**Figure 4:**
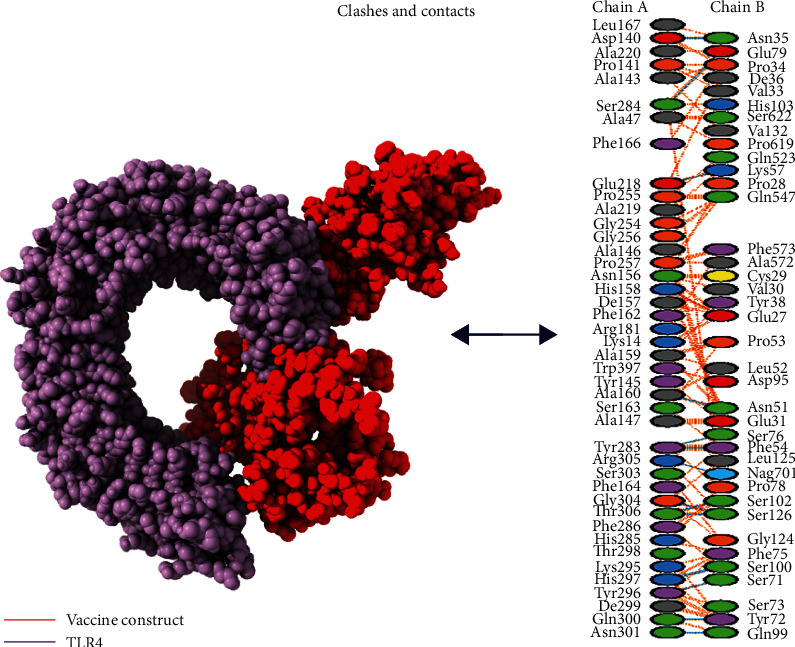
Docked complex of TLR4 construct with the vaccine construct indicating the interacting part along with the interacting residue. The red part is for the vaccine, and the purple is for theTLR4 in this docking.

**Figure 5:**
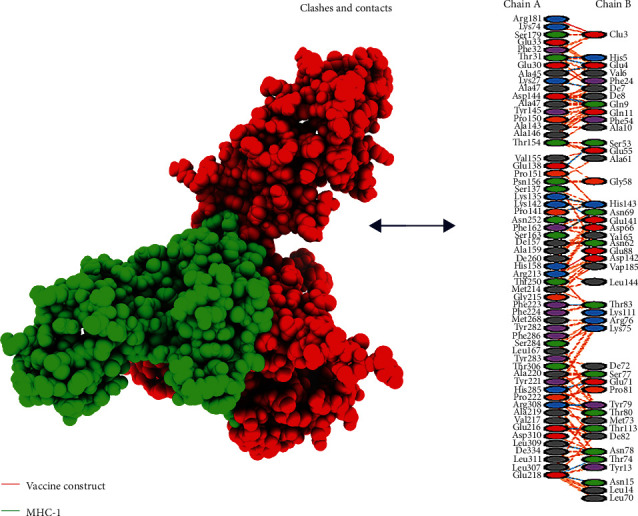
Interaction of vaccine construct with MHC-I construct. The red part is of the vaccine, and the green one is indicating the MHC-I construct; clashes and contacts are also given.

**Figure 6:**
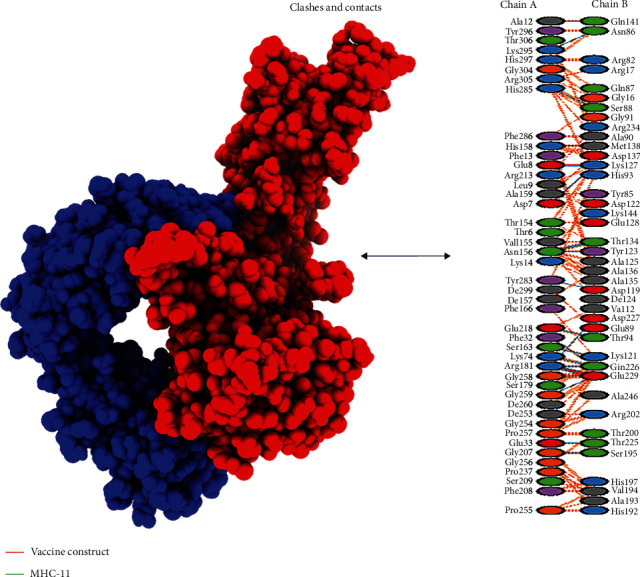
Interaction of vaccine construct with MHC-II construct. The blue part depicts the MHC-II and clashes, and contacts are given on the right side of the figure.

An active immune response requires effective antigen-receptor interaction. Using HADDOCK v.2.4, the vaccine was docked with TLR4, MHC-I, and MHC-II receptors (immune receptors of humans). TLR4 facilitates an effective immune response to bacterial recognition. Vaccine and TLR4 show a substantial interaction, according to the docking data. The TLR4 vaccine binding score was calculated to be 635.2 kcal/mol. On the map, TLR4 was shown in cyan, whereas MEBSV was shown in green (Figure 4). It was discovered that TLR4 and vaccination had desolvation energy within a range of -51.1 kcal^−1^. With the use of the HADDOCK v.2.4 software, the vaccine structure was also docked with the MHC-I and MHC-II receptors. HADDOCK scores of 345.4 and 279.8 were found, respectively, with desolvation energies of -43.3 kcal^−1^ and -67.6 kcal^−1^. The docking results are displayed in Table 5. Eleven (11) hydrogen bond interactions were discovered in the docked complex of MHC-II and vaccine design. However, there were seven interacting residues in the MHC-I complex, as illustrated in Figures 5 and 6.

### 3.10. Normal Mode Analysis

**Figure 7:**
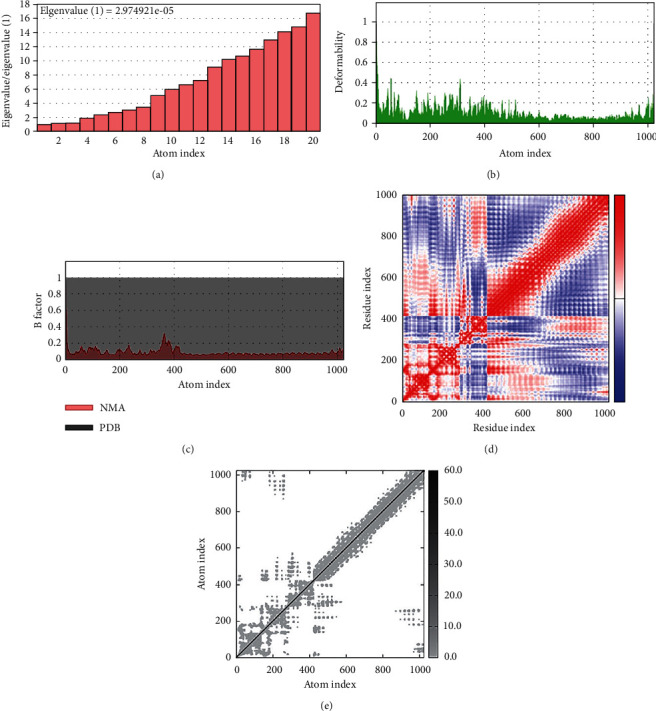
(a–e) Molecular dynamic simulation of the vaccine-TLR4 complex, showing (a) eigenvalue, (b) deformability, (c) *B*-factor, (d) covariance matrix, and (e) elastic network analysis.

The normal mode analysis (NMA) was utilized to explore the mobility and stability of proteins on a wide scale. The iMODS server was employed for it, which relied on the internal coordinates of the docked complex. Individual residue distortion determined the complex's deformability, as evidenced by the chain's hinges (Figure 7(b)). The value for the complex was calculated as 2.974921*e* − 05 (Figure 7(a)). The eigenvalue was calculated by inverting the variance associated with each normal mode [79]. As a consequence of the normal mode analysis, the *B*-factor value was proportional to RMS (Figure 7(c)). A covariance matrix represents the pairings of residue pairs, with different colors representing associated, disassociated, or irrelevant movements, such as red, blue, and white (Figure 7(d)). The elastic map showed spring-connected joint atoms, with each point representing one spring and a grey color indicating stiffer locations, with intensity proportional to stiffness (Figure 7(e)). These results have been evaluated for TLR4 because it is a universal human receptor to compare any results through it [80].

### 3.11. Immune Simulation

**Figure 8:**
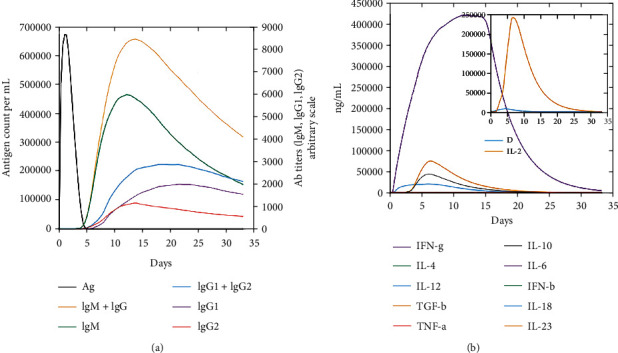
(a, b) In silico immune responses of vaccine as an antigen: (a) immunoglobulin generation and B cell isotypes following exposure in different states with the Simpson index to the antigen; (b) development of cytokine and interleukins.

Every secondary and primary immune response contributes to distinct immunological responses to diseases. The host immune system responds to the antigen *in silico* as shown in Figure 8. The results of the simulations of the candidate vaccine were found to be very similar. Both the candidate vaccine and the control showed a significant drop in the number of antigens over time. After the second and third doses of the candidate vaccine, the levels of antibodies were much higher. There was a rise in IgG+IgM and a fall in antigen in the secondary and primary stages of the initial reaction, which was characterized by high levels of IgG+IgM and IgM. The effectiveness of interleukin and cytokine reactions has been discovered as indicated; there was an effective immunological response to the vaccine, as well as clearance after consecutive treatments (Figure 8).

### 3.12. In Silico Cloning

**Figure 9:**
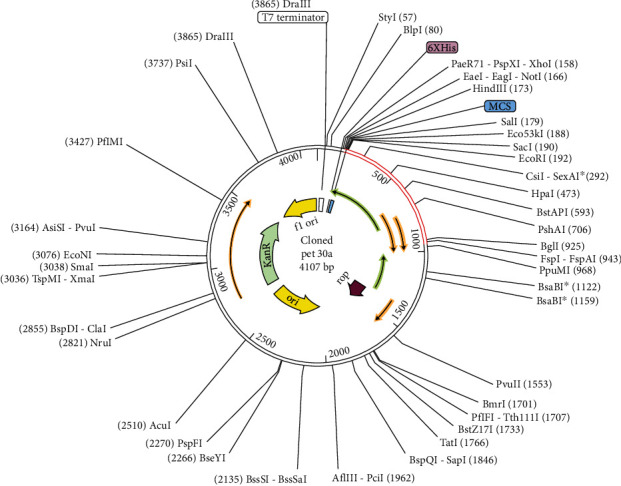
In silico cloning of codon-optimized vaccine into E. coli K12 expression system. The plasmid backbone is kept in black while the inserted DNA sequence is shown in red color.

Codon optimization and *in silico* cloning were utilized to validate that the vaccine protein was successfully produced in the *E. coli* host system. The vaccine's codons are identical to the *E. coli* K12 codon of the potential host. First, the vaccine sequence was reversed and transcribed to make the cDNA of the vaccine, and then, some changes have been done if needed to enhance the GC contents of the sequence and CAI value. In enhanced DNA, the CAI value was 0.9, and the GC content value was 48.87 percent. The synthesized codon was inserted into the E. coli vector pET 30a (+) between Nco1 and restriction sites. Additionally, the clone was 6452 bp in length (Figure 9).

## 4. Discussion

Despite advancements in the treatment of infectious diseases, pathogenic microorganisms continue to pose the greatest threat to public health, while conventional vaccines have largely been responsible for the treatment or eradication of some pathogens, the rapid emergence of infectious diseases necessitates new approaches to vaccine development [81]. Traditional vaccine production techniques require the cultivation of pathogenic microorganisms and identifying their immunogenic components, which takes time and can only detect antigens that are highly produced and purified [82]. Protein abundance does not always imply immunogenicity, and antigens produced *in vivo* during pathogenesis cannot always be produced in *in vitro* conditions. Possible surface proteins were identified using a strategy that begins with the genome rather than the microbe and uses computational tools and pattern recognition to identify them [83]. As a result, in addition to finding all antigens that can be investigated using traditional approaches, this method can also identify novel antigens critical to new vaccines' immunogenicity. By focusing on *in silico* investigations, vaccine design and manufacture can be completed in the quickest possible time [84]. In reality, this form of vaccine creation is a comprehensive and successful example of computer-aided biotechnology, which was originally utilized to overcome the limitations of traditional methods [85]. Based on these ideas, “immune informatics,” a bioinformatics approach focusing on immunology and vaccination, has arisen. Immunoinformatics is currently regarded as a powerful example of applied bioinformatics in the field of immunology. A reverse vaccinology design demonstrates how immune informatics may help and validate biotechnological research [86].

The amino acid sequences of antigens were downloaded from NCBI for the first phase in the current investigation to build a multiepitope vaccine against *Legionella pneumophila*. Then, using an immune informatics technique, possible CTL, HTL, and LBL epitopes were predicted and examined. Multi T cell epitope subunit vaccinations are currently gaining popularity. Subunit epitope vaccines have several benefits, including low cost, high specificity, and safety. Some bioinformatics and immune informatics technologies have been created in recent years that are useful in vaccine design. Recognizing immunogenic epitopes from protective antigens is critical for developing epitope-based subunit vaccines. Immunoinformatics techniques can find random/indiscriminate immune-dominant epitopes from any protein, saving time and money while also assisting in identifying appropriate epitopes.

Epitopes of CTL, HTL, and B cell were predicted to develop the construct vaccine based on their immunogenicity and antigenicity. Helper T cells that deliver cytokines including interleukin-4 (IL-4), interferon-gamma (IFN-*γ*), and interleukin-10 (IL-10) may minimize tissue damage by preventing proinflammatory responses. Cytotoxic T cells, B lymphocytes, the innate immune system, and other immune cells are all stimulated by helper T cells. As a result, during the selection of the fusion construct vaccine, the capacity of specific HTL epitopes to induce cytokine was also evaluated. GPGPG, AAY, and KK linkers were used to bind HTL, CTL, and B cell epitopes, respectively, to finish the vaccine assembly. In developing vaccine peptides, linkers can help in expression, stability, and folding. The vaccine we produced has a molecular weight of 46284.37 kDa in this research, which falls in the normal range of a multi-epitope-based vaccine's molecular weight. The solubility of overexpressed recombinant proteins within an *E. coli* host is crucial in functional and biochemical investigations [87]. By comparing these results with the previously published papers, we found that results of our research work were similar according to the standards [72, 88]. The produced vaccine protein's solubility was observed, indicating that it has simple access to the host. The theoretical pI value was determined to validate the vaccine's potential, exhibiting basic nature. Moreover, the stability index forecast in our study validates the protein's stability following expression, representing an increase in its usage capacity. The aliphatic index and GRAVY score, respectively, measure thermostability and hydrophilicity [89]. The 3D structure of a protein provides information on the protein's unique assembly and helps understand protein dynamics and protein-ligand interactions of other proteins. The vaccine's desired properties were significantly improved once it was refined.

Only a few residues were found in the prohibited zone by the Ramachandran plot, whereas the majority were found in preferred regions (92.9 percent). This further confirmed the entire model's adequacy in terms of quality. Additionally, energy minimization was used to minimize the system's potential energy and stabilize the entire structure of the multiepitope vaccine. Molecular dynamic simulation and ligand-receptor docking studies were used to assess the stability and potential immunological interaction between multiepitope vaccination and TLR4, MHC-I, and MHC-II as adjuvant. Serological testing of a vaccine's immunological function is one of the preliminary steps in its certification [90]. Immune simulation was performed to validate the immunity response of the body after injecting the constructed vaccine, and the results were compared to standards mentioned in previous publications [72]. A suitable host must be used to express the recombinant protein. To make recombinant proteins, E. coli expression methods were used. In this study, codon optimization was aimed at achieving high-level recombinant vaccine protein expression in *E. coli* K12. The obtained values for GC content (50.51 percent) and codon adaptation are supposed to predict protein expression in bacteria (0.97). Improving protein stability in many mechanical and biological applications is the primary goal. This research employed immune informatics to create a new multiepitope vaccination against *L. pneumophila* that potentially induces immunological responses mediated by cells and humoral immune responses.

## 5. Conclusions

*L. pneumophila* is a global health threat as medication or vaccinations are still ineffective in treating or preventing it. Antibacterial drugs have been studied, but none are efficient in avoiding its infection. Using subtractive genomics and immunoinformatics, the main goal of making a MEBS vaccine is to control the body's humoral and cellular immune responses. Using subtractive genomics, the therapeutic proteins needed for bacterial survival but not found in the host were found. The proposed MEBSV model, when combined with computer analyses and immune-information data, could lead to the development of a possible vaccine against *L. pneumophila*. Further research is needed to establish the MEBS vaccine model's efficiency and safety.

## Figures and Tables

**Table 1:** Top selected proteins with highest antigenic nature with extracellular location.

Sr. no.	Protein name	Accession no.	Antigenicity	Helices	Location
1	YARHG domain-containing protein	A0A127V420	0.51	0	Extracellular
2	TPR-region domain-containing protein	A0A140J7Y2	0.72	0	Extracellular
3	SPOR domain-containing protein	A0A140J8J4	0.69	0	Extracellular
4	Sel1 repeat protein	A0A127V446	0.54	0	Extracellular
5	Metalloprotease PmbA	A0A127V344	0.55	0	Extracellular
5	Peptide ABC transporter substrate-binding protein	A0A140J2V6	0.53	0	Extracellular

**Table 2:** Final CTL selected epitopes for the construction of vaccine against *L. pneumophila*.

Epitope	Protein	Alleles	Position	Antigenicity	Immunogenicity
CSEIDPKADY	YARHG domain-containing protein	HLA-A∗01:01 HLA-A∗30:02	4-35	1.4910	0.0202
FPPLATVNIH	YARHG domain-containing protein	HLA-B∗53:01 HLA-B∗35:01	3-38	1.1473	0.1723
FSFLFLFSTFTF	Uncharacterized protein	HLA-A∗23:01 HLA-A∗24:02 HLA-B∗46:01 HLA-B∗15:02 HLA-A∗29:02 HLA-C∗07:02 HLA-B∗58:01 HLA-B∗35:01 HLA-B∗57:01	1-6	1.8775	0.24096
NVSGRTLRLDLW	Uncharacterized protein	HLA-B∗58:01	1-49	2.4487	0.11
KPQWLLSLGYEY	SPOR domain-containing protein	HLA-A∗29:02 HLA-A∗30:02 HLA-B∗35:01	6-27	1.1161	0.07907
GFSVDVRMGEVE	Metalloprotease PmbA	HLA-B∗46:01 HLA-C∗08:02 HLA-C∗15:02	1-47	1.9821	0.06956
PFFAPVPWEADL	Peptide ABC transporter substrate-binding protein	HLA-B∗35:03 HLA-C∗03:03 HLA-C∗07:02	5-10	1.0915	0.4519

**Table 3:** Final HTl epitopes for the construction of vaccine against Legionella pneumophila.

Epitope	Protein	Alleles	Position	Antigenicity	IFN-*γ*	IL-4	IL-10
YFWKTTFPPLATVNI	YARHG domain-containing protein	HLA-DPA1∗02:01/DPB1∗14:01	152-166	0.8325	Positive	Inducer	Negative
GIYYSHFFNMDINGQ	SPOR domain-containing protein	HLA-DRB1∗04:05 HLA-DPA1∗01:03/DPB1∗04:01	161-175	1.1525	Positive	Inducer	Negative
VGIYYSHFFNMDING	SPOR domain-containing protein	HLA-DRB1∗04:05 HLA-DPA1∗01:03/DPB1∗04:01 LA-DPA1∗01:03/DPB1∗02:01	160-174	1.0893	Positive	Inducer	Negative

**Table 4:** Final selected B cell epitopes for vaccine construction.

Epitope	Protein	Score	Position	Antigenicity	Immunogenicity
YHTIQNVSGRTLRLDL	Uncharacterized protein	0.85	44	1.5483	0.06924
GRTLRLDLWVDKIVAG	Uncharacterized protein	0.73	52	1.1531	0.30232
LFIITLNLHCINVAFS	SPOR domain-containing protein	0.73	13	1.6755	0.4296
SWDITPQQAIDLALKC	Metalloprotease PmbA	0.78	131	1.5251	0.02249
KNHPDLDLYHSWDITP	Metalloprotease PmbA	0.78	121	1.0760	0.25932
KMRIGHAQIFSWGWNA	Peptide ABC transporter substrate-binding protein	0.92	550	1.2577	0.65378
DLNIRATLYNRFQEKM	Peptide ABC transporter substrate-binding protein	0.62	536	1.2516	0.25128

**Table 5:** Docking table indicating the docking scores along with different energy values of TLR4, MHC-I, and MHC-II with vaccine construct.

Parameters	Values
TLR4	
HADDOCK v.2.2 score	635.2 ± 9.3
Cluster size	18
RMSD from the overall lowest energy structure	21.3 ± 0.0
Van-der-Waals energy	−130.5 ± 14.2
Electrostatic energy	−352.4 ± 71.9
Desolvation energy	−51.1 ± 12.3
Restraint violation energy	3412.9 ± 178.99
Buried surface area	3918.1 ± 163.1
*Z*-score	-1.8
MHC-I receptor	
HADDOCK v.2.4 score	345.3 ± 27.1
Cluster size	11
RMSD from the overall lowest energy structure	16.7 ± 0.0
Van-der-Waals energy	−136.6 ± 10.0
Electrostatic energy	−324.3 ± 32.8
Desolvation energy	−43.3 ± 5.6
Restraint violation energy	3678.0 ± 162.5
Buried surface area	4565.7 ± 289.0
*Z*-score	-1.3
MHC-II receptor	
HADDOCK v.2.4 score	279.8 ± 31.5
Cluster size	9
RMSD from the overall lowest energy structure	17.3 ± 0.4
Van-der-Waals energy	−148.5 ± 15.4
Electrostatic energy	−289.3 ± 39.0
Desolvation energy	−67.6 ± 5.1
Restraint violation energy	3106.9 ± 165.4
Buried surface area	3781.5 ± 267.0
*Z*-score	-1.6

## Data Availability

All available data are included in the manuscript.

## Supplementary Materials

Supplementary Materials The data used to support the findings of this study are included within Supplementary Materials. Table S1: final MHC-I epitopes selected on the basis of different parameters. Table S2: final MHC-II epitopes selected on the basis of different parameters. Table S3: final B cell epitopes selected on the basis of different parameters.
