# Supplementary material for: Vaccinomics to Design a Multiepitope Vaccine against Legionella pneumophila
Source: Biomed Res Int. 2022 Sep 17;2022:4975721. doi: 10.1155/2022/4975721 (PMC9509222; doi:10.1155/2022/4975721)
Supplement: Supplementary Materials — The data used to support the findings of this study are included within Supplementary Materials. Table S1: final MHC-I epitopes selected on the basis of different parameters. Table S2: final MHC-II epitopes selected on the basis of different parameters. Table S3: final B cell epitopes selected on the basis of different parameters. [file 4975721.f1.pdf]

Table S 1: Final MHC-I epitopes selected on the basis of different parameters

Protein	Epitopes	Position	alleles	antigenicity	immunogenicity
YARHG domain-containing protein	KYIWDLRHF	5-7	HLA-A*23:01 HLA-A*24:02	0.6046	0.33983
	FPPLATVNI	3-38	HLA-B*51:01 HLA-B*53:01	0.9477	0.09094
	SNKELRFLR	5-49	HLA-A*33:01 HLA-A*31:01 HLA-A*68:01	0.5345	0.16099
	CSEIDPKADY	4-35	HLA-A*01:01 HLA-A*30:02	1.4910	0.0202
	FPPLATVNIH	3-38	HLA-B*53:01 HLA-B*35:01	1.1473	0.1723
TPR-REGION domain-containing protein	QLKAFSWSDLWF	1-17	HLA-B*57:01 HLA-B*58:01 HLA-A*32:01	0.5811	0.11781
	MMWRVLAIVA	1-1	HLA-A*02:01	0.5143	0.32665
Uncharacterized protein	FSFLFLFSTFTF	1-6	HLA-A*23:01 HLA-A*24:02 HLA-B*46:01 HLA-B*15:02	1.8775	0.24096

			HLA-A*29:02 HLA-C*07:02 HLA-B*58:01 HLA-B*35:01 HLA-B*57:01		
	CLFSFLFLFSTF	1-4	HLA-B*46:01 HLA-A*23:01 HLA-A*24:02 HLA-A*29:02	1.7732	0.07119
	SFLFLFSTFTFS	1-7	HLA-A*23:01 HLA-A*24:02 HLA-B*15:02 HLA-C*07:02	1.2515	0.30546
	LFSFLFLFSTFT	1-5	HLA-B*46:01 HLA-A*23:01 HLA-A*24:02	1.6839	0.21593
	FLFLFSTFTFSQ	1-8	HLA-A*23:01 HLA-B*15:02 HLA - A*24:02 HLA-C*07:02	1.2934	0.17022
	LFLFSTFTFSQN	9-20	HLA-A*23:01 HLA-A*24:02	0.7424	0.0456
	NVSGRTLRLDLW	1-49	HLA-	2.4487	0.11

			B*58:01		
SPOR domain-containing protein	MFRINLKRYVAL	1-1	HLA-B*08:01 HLA-C*06:02 HLA-B*39:01 HLA-C*07:01	0.6512	0.02824
	KRYVALLFIITL	1-7	HLA-B*27:05 HLA-A*23:01 HLA-A*24:02	0.9141	0.43112
	LPEFTNYNYQWK	5-1	HLA-B*53:01	0.6270	0.20008
	KPQWLLSLGYEY	6-27	HLA-A*29:02 HLA-A*30:02 HLA-B*35:01	1.1161	0.07907
Sel1 repeat protein	GFYWYLNTPEGY	2-36	HLA-A*29:02 HLA-C*14:02 HLA-A*30:02 HLA-B*15:02 HLA-C*07:02	1.0450	0.33265
	NVDAQVLLAGFY	2-27	HLA-A*01:01 HLA-A*30:02 HLA-A*26:01	0.7063	0.0862
	AQFNLADMYFYG	5-29	HLA-A*30:02 HLA-A*29:02	0.7846	0.0862
	VAADPVKAYAWF	6-29	HLA-C*05:01 HLA-	0.6489	0.05148

			B*58:01		
Metalloprotease PmbA	GFSVDVRMGEVE	1-47	HLA-B*46:01 HLA-C*08:02 HLA-C*15:02	1.9821	0.06956
	FSVDVRMGEVET	1-48	HLA-B*46:01 HLA-C*08:02 HLA-C*15:02	1.7369	0.17591
	YHSWDITPQQAI	3-9	HLA-B*38:01 HLA-B*39:01	0.9313	0.16345
	HPDLDLYHSWDI	3-3	HLA-B*53:01	1.1002	0.07891
	NHPDLDLYHSWD	3-2	HLA-B*53:01 HLA-B*38:01	0.6182	0.03537
Peptide ABC transporter substrate-binding protein	FAPVPWEADLFY	5-12	HLA-B*35:03 HLA-B*35:01 HLA-B*53:01 HLA-A*29:02 HLA-C*03:03 HLA-B*46:01	0.5302	0.45836
	PFFAPVPWEADL	5-10	HLA-B*35:03 HLA-C*03:03 HLA-C*07:02	1.0915	0.4519
	RRIWNKPVFWPL	12-15	HLA-B*27:05 HLA-A*32:01 HLA-	**0.5926**	**0.36985**

			B*48:01 HLA-C*07:02 HLA-B*08:01 HLA-A*02:06 HLA-B*39:01		
	RRIWNKPVFWPL	12-15	HLA-A*32:01	0.5926	0.36985

Table S 2: Final MHC-II epitopes selected on the basis of different parameters

protein	SN	HTL epitopes	Position	alleles	Antigenicity	IFN-Y	IL-4	IL-10
YARHG domain-containing protein	1	DAYFWKTTFPPLATV	150-164	HLA-DPA1*01:03/DPB1*04:01	0.5734	positive	IL-4 inducer	negative
	2	AYFWKTTFPPLATVN	151-165	HLA-DPA1*01:03/DPB1*04:01 HLA-DPA1*02:01/DPB1*14:01	0.6557	positive	IL-4 inducer	negative
	3	DAYFWKTTFPPLATV	204-218	HLA-DPA1*02:01/DPB1*14:01 HLA-DPA1*02:01/DPB1*01:01 HLA-DPA1*02:01/DPB1*05:01	0.5734	positive	IL-4 inducer	negative
	4	YFWKTTFPPLATVNI	152-166	HLA-DPA1*02:01/DPB1*14:01	0.8325	Positive	IL-4 inducer	negative
SPOR domain-contain	1	GIYYSHFFNMDINGQ	161-175	HLA-DRB1*04:05 HLA-DPA1*01:03/DPB1*04:01	1.1525	positive	IL-4 inducer	Negative

ing protein	2	VGIYYSHFFNMDING	160-174	HLA-DRB1*04:05 HLA-DPA1*01:03/DPB1*04:01 LA-DPA1*01:03/DPB1*02:01	1.0893	positive	IL4-inducer	Negative

Table S 3: Final B-cell epitopes selected on the basis of different parameters

Protein	B cell Epitope	Position	Score	Antigenicity
YARHG domain-containing protein	DGSIGHTRLEVIPNTP	196	0.92	0.8371
	LIRISGRNLNEPHMNG	54	0.80	0.7503
Uncharacterized protein	GLAIIADYPYVNGVTG	101	0.91	0.5921
	YHTIQNVSGRTLRLDL	44	0.85	1.5483
	VDKIVAGTANVQSDNV	61	0.78	0.7981
	GRTLRLDLWVDKIVAG	52	0.73	1.1531
	FSFLFLFSTFTFSQNF	6	0.69	1.4032
	DYPYVNGVTGQFSIHY	107	0.52	0.7611
SPOR domain-containing protein	IGGQETTPDDQFTVNN	164	0.92	1.1461
	HTTTVFAYQLGAGLDW	309	0.78	0.7868
	LFIITLNLHCINVAFS	13	0.73	1.6755
	SHFFNMDINGQVIQYS	225	0.51	1.0093
Sel1 repeat protein	DGIGVAADPVKAYAWF	315	0.83	0.8210
	AGFYWYLNTPEGYKKA	95	0.77	0.8607
	QGNSNAALAIGYNYDT	156	0.70	0.9605
Metalloprotease	MQRDYDYTTARNAENL	203	0.85	0.6989

PmbA	HNLTINPTCGDLGDLL	351	0.83	0.7878
	KGALGSAPFDGEGVPT	300	0.80	0.5064
	SWDITPQQAIDLALKC	131	0.78	1.5251
	KNHPDLDLYHSWDITP	121	0.78	1.0760
	PFDGEGVPTRPNVLVE	307	0.76	0.7194
	AVNYDRGFSVDVRMGE	41	0.72	1.0010
	AENLVAAELIAAKAVE	215	0.55	0.5576
Peptide ABC transporter substrate-binding protein	KMRIGHAQIFSWGWNA	550	0.92	1.2577
	PPGIFGYKEGKEGINP	451	0.92	0.6111
	MVEAGYPGGIDPKTGK	488	0.87	1.3027
	WGWNADYPDPENFLFL	561	0.84	0.5314
	PVFWPLGVLFGVLLAL	681	0.81	0.7898
	DMSGISADSFDQAIHI	357	0.77	0.8478
	YLDAQGNPVPAGDNTK	90	0.74	0.6330
	DFKYTGTRELIIDDYI	155	0.72	0.6941
	YKRPYELIPLTATWLP	71	0.67	0.5218
	DLNIRATLYNRFQEKM	536	0.62	1.2516
	NKRARKLRQAISIAVN	415	0.58	0.9616
	VLLALVIPLFIAYLKK	692	0.57	1.1225
